# Neuroprotective Effects of Ethanol Extract of *Polyscias fruticosa* (EEPF) against Glutamate-Mediated Neuronal Toxicity in HT22 Cells

**DOI:** 10.3390/ijms24043969

**Published:** 2023-02-16

**Authors:** Baskar Selvaraj, Tam Thi Le, Dae Won Kim, Bo Hyun Jung, Ki-Yeon Yoo, Hong Ryul Ahn, Phuong Thien Thuong, Thi Thu Thuy Tran, Ae Nim Pae, Sang Hoon Jung, Jae Wook Lee

**Affiliations:** 1 Natural Product Research Center, Institute of Natural Product, Korea Institute of Science and Technology, Gangneung 25451, Republic of Korea; 2 Division of Bio-Medical Science & Technology, University of Science and Technology (UST), Daejeon 34113, Republic of Korea; 3 Department of Biochemistry and Molecular Biology, Research Institute of Oral Science, College of Dentistry, Gangneung Wonju National University, Gangneung 25457, Republic of Korea; 4 Department of Anatomy, College of Dentistry, Gangneung Wonju National University, Gangneung 25457, Republic of Korea; 5 Division of Biotechnology, Vietnam Korea Institute of Science and Technology, Hoa Lac High-Tech Park, km29 Thang Long Boulevard, Hanoi 13113, Vietnam; 6 Institute of Natural Products Chemistry, Vietnam Academy of Science and Technology, 18 Hoang Quoc Viet, Caugiay, Hanoi 13113, Vietnam; 7 Department of Chemistry, Graduate University of Science and Technology, VAST, 18 Hoang Quoc Viet, Caugiay, Hanoi 13113, Vietnam; 8 Center of Brain Disorders, Brain Science Institute, Korea Institute of Science and Technology, Seoul 02792, Republic of Korea

**Keywords:** HT22 cells, *Polyscias fruticosa*, neuroprotection, reactive oxygen species, ischemia

## Abstract

In traditional herbal medicine, the *Polyscias fruticosa* has been frequently used for the treatment of ischemia and inflammation. Oxidative stress mediated by elevated glutamate levels cause neuronal cell death in ischemia and various neurodegenerative diseases. However, so far, the neuroprotective effects of this plant extract against glutamate-mediated cell death have not been investigated in cell models. The current study investigates the neuroprotective effects of ethanol extracts of *Polyscias fruticosa* (EEPF) and elucidates the underlying molecular mechanisms of EEPFs relevant to neuroprotection against glutamate-mediated cell death. The oxidative stress-mediated cell death was induced by 5 mM glutamate treatment in HT22 cells. The cell viability was measured by a tetrazolium-based EZ-Cytox reagent and Calcein-AM fluorescent dye. Intracellular Ca^2+^ and ROS levels were measured by fluorescent dyes, fluo-3 AM and 2′,7′-dichlorodihydrofluorescein diacetate (DCF-DA), respectively. Protein expressions of p-AKT, BDNF, p-CREB, Bax, Bcl-2, and apoptosis-inducing factor (AIF) were determined by western blot analysis. The apoptotic cell death was measured by flow cytometry. The in vivo efficacy of EEPF was evaluated using the Mongolian gerbil mouse by surgery-induced brain ischemia. EEPF treatment showed a neuroprotective effect against glutamate-induced cell death. The EEPF co-treatment reduced the intracellular Ca^2+^ and ROS and apoptotic cell death. Furthermore, it recovered the p-AKT, p-CREB, BDNF, and Bcl-2 levels decreased by glutamate. The EEPF co-treatment suppressed the activation of apoptotic Bax, the nuclear translocation of AIF, and mitogen-activated protein kinase (MAPK) pathway proteins (ERK1/2, p38, JNK). Further, EEPF treatment significantly rescued the degenerative neurons in the ischemia-induced Mongolian gerbil in vivo model. EEPF exhibited neuroprotective properties that suppress glutamate-mediated neurotoxicity. The underlying mechanism of EEPF is increasing the level of p-AKT, p-CREB, BDNF, and Bcl-2 associated with cell survival. It has therapeutic potential for the treatment of glutamate-mediated neuropathology.

## 1. Introduction

The traditional medicinal herb *Polyscias fruticosa* is a perennial plant that grows in large tropical areas of Asian countries such as Vietnam and other tropical Pacific regions. *Polyscias* belongs to the Araliaceae family and is one of the largest genera consisting of 159 species. Among them, seven species are widely distributed in Vietnam [1,2,3]. *P. fruticosa* root extracts are used as diuretics, antipyretics, and anti-dysentery remedies, as well as for pain relief treatment of rheumatism and neuralgia. *P. fruticosa* extracts have been reported to contain saponins, polyphenols, flavonoids, and vitamins which have various effects including antioxidant activity [4]. Therefore, the leaf extracts of *Polyscias fruticosa* are used extensively for their antioxidant [5] and anti-inflammatory [6] properties; moreover, they are used as medicine for asthma [7] and osteoclasts [8]. It is also widely used for the treatment of inflammation and ischemia. In particular, *P. fruticosa* has been grown widely in Vietnam for herbal medicinal applications, especially due to its increased blood flow effect on the brain.

Oxidative stress imbalance is one of the major causes of neurodegenerative diseases. Neuronal cells are highly vulnerable to oxidative stress because of the high metabolism of oxygen consumption, and it is prone to generate high levels of ROS [9]. An abnormal glutamate release is associated with the progression of neurodegeneration and ischemic brain damage [10]. HT22 cells, derived by immortalization of mouse hippocampal neurons, lack functional ionotropic glutamate receptors. However, they are sensitive to excessive glutamate, which causes depletion of glutathione and endogenous antioxidants, eventually leading to neuronal cell death. Therefore, HT22 cells are an appropriate in vitro model for studying oxidative stress-induced neuronal toxicity [11].

High glutamate treatment leads to impairment of mitochondrial function due to abnormal changes in Ca^2+^ homeostasis [12,13]. Glutamate toxicity also alters the expression of pro-and anti-apoptotic proteins such as Bax and Bcl-2. Excessive glutamate disrupts the mitochondrial membrane potential, leading to the activation of apoptosis via the release of cytochrome C and the nuclear translocation of apoptosis-inducing factor (AIF) [14,15,16]. The generation of ROS caused by intracellular oxidative stress activates the mitogen-activated protein kinase (MAPK) signal pathway [17], which is strongly associated with inflammation, differentiation, and apoptosis pathways [18]. Oxidative stress activates MAPK proteins via the phosphorylation of JNK, ERK1/2, and p38. These proteins affect differentiation and cell death pathways during various stress conditions such as heat shock, and UV exposure [19,20].

The phosphatidylinositol-3 kinase (PI3K)/AKT pathway plays a crucial role in maintaining neuronal cell survival signals [21,22,23]. Under neuroprotective conditions, AKT activates the transcription factor CREB (CRE-binding protein) via phosphorylation [24,25]. CREB highly regulates the expression of brain-derived neurotrophic factors (BDNF), which are associated with the inhibition of MAPKs and apoptotic pathways [26]. Recent studies have shown that some natural products activate the AKT and CREB pathways that suppress oxidative stress-induced cell death [27].

Cerebral ischemia causes irreversible neuronal loss by inducing various pathological insults such as ROS, oxidative stress, and inflammatory cytokines. The toxic stress signaling induced by elevated ROS and oxidative stress seems to further aggravate neuronal damage by inducing cell death pathways. Therefore, finding natural compounds with anti-oxidant potential can be a promising therapeutic strategy [28].

Herein, we have investigated the *P. fruticosa* extracts and elucidated the molecular mechanisms underlying the neuroprotective effects in murine hippocampal neuronal cells and further confirmed its effect in a surgery-induced ischemic stroke model using Mongolian gerbil mice.

## 2. Results

### 2.1. Neuroprotection of P. fruticosa Extracts against Glutamate-Induced Cell Death

**Figure 1:**
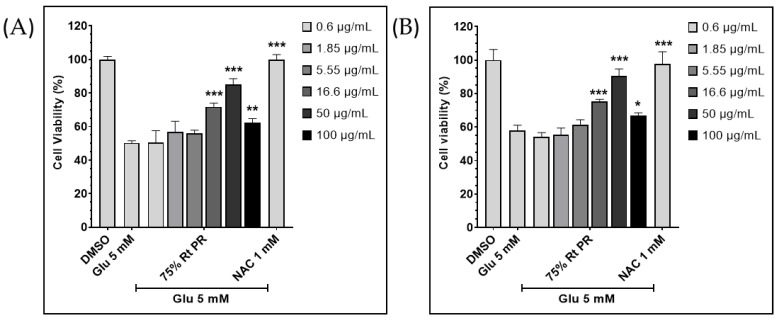
The 75% EEPF prevents glutamate induced neuronal cell death. (**A**) Relative cell survival of HT22 hippocampal neuronal cell after treatment with 5 mM of glutamate (Glu) in the presence or absence of 75% EEPF. Calcein-AM stained live cells were counted by Operetta image analysis system. (**B**) Relative cell survival of HT22 hippocampal neuronal cells after treatment with 5 mM of glutamate in the presence and absence of 75% EEPF. Tetrazolium based EZ-Cytox reagent was used for measuring cell viability, NAC; n-acetylcysteine. Data bar graphs are presented as means ± SD, n = 4, and the *p* values *** *p* > 0.001, ** *p* > 0.01, * *p* > 0.05.

The different samples of extracts were prepared from the roots and leaves of *P. fruticosa* (PF) by changing the combination of ethanol and water percentage. To evaluate the neuroprotection, we used a high concentration of glutamate, 5 mM on HT22 cells to select a more potent *Polyscias* root extract. The root extracts with various ethanol-water mixture solvents differentially inhibited glutamate-induced cell death; however, the 75%-ethanol PF root extract significantly prevented glutamate-induced cell death at 50 µg/mL (Figure S1). The 75%-ethanol PF root extract was named 75% EEPF. We further confirmed its protective effects against glutamate using two different assays with different concentrations. The calcein-AM fluorescent dye and tetrazolium-based EZ-Cytox (Dogen bio, Seoul, Korea) assays significantly supported its neuroprotection effect (Figure 1A,B). Therefore, we selected 75% of EEPF for further investigation to study the downstream signaling events leading to neuroprotection.

### 2.2. The 75% EEPF Prevents ROS Generation and Ca^2+^ Influx

**Figure 2:**
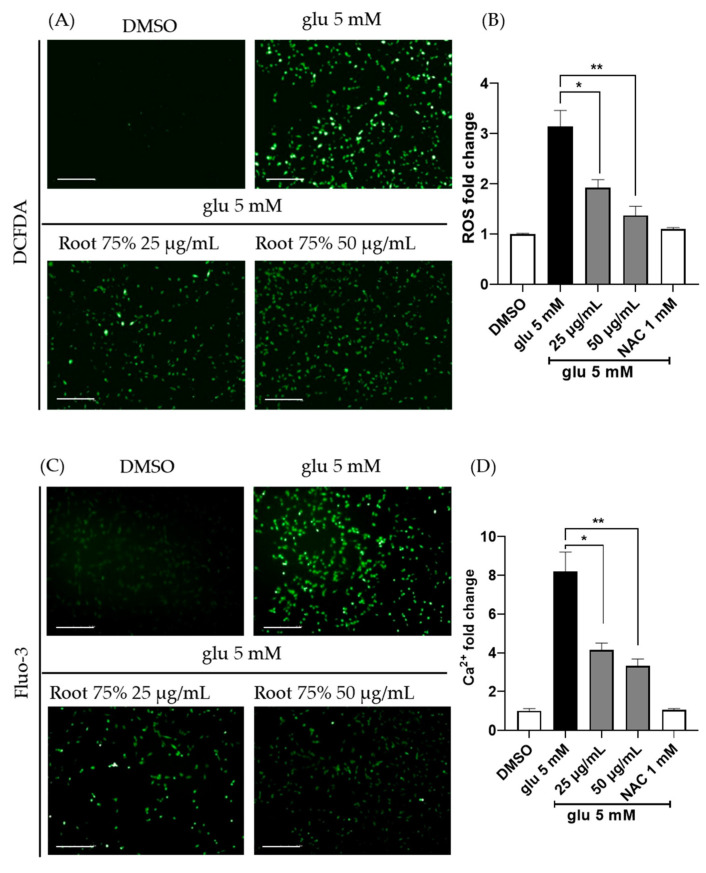
The 75% EEPF root prevents intracellular ROS and Ca^2+^ ion generation induced by glutamate. (**A**) Fluorescent image analysis of cellular ROS using DCFDA (scale bar = 100 μm). (**B**) An amount of 5 mM of glutamate treatment increased ROS levels in HT22 cells, but 75% EEPF root extract has significantly suppressed ROS generation. *p* values of * *p* < 0.02, ** *p* < 0.0075. (**C**) Fluorescent image analysis of cellular Ca^2+^ ion concentration using Fluo-3 dye, a calcium sensor (scale bar = 100 μm). (**D**) An amount of 5 mM of glutamate increased cellular Ca^2+^ ion levels, but the co-treatment of 75% EEPF prevented the Ca^2+^ ion accumulation. Data bar graphs are presented as means ± SD, n = 3, *p* values of * *p* < 0.014, ** *p* < 0.0083.

A high concentration of extracellular glutamate increases intracellular ROS levels and Ca^2+^ influx. Therefore, we investigated the effect of 75% EEPF. Intracellular ROS levels were measured using DCF-DA dye. Glutamate treatment enhanced ROS levels inside the cells, but co-treatment with 75% EEPF prevented ROS generation (Figure 2A,B). This indicates that neuroprotection by this extract is associated with the inhibition of ROS signaling.

Abnormal Ca^2+^ influx is also induced by glutamate which leads to the accumulation of ROS and oxidative stress; therefore, we studied Ca^2+^ influx with Fluo-3-AM fluorescent dye. Co-treatment with 75% EEPF inhibited glutamate-mediated Ca^2+^ accumulation (Figure 2C,D). These data show that 75% EEPF significantly suppressed the toxic ROS and Ca^2+^ influx levels induced by glutamate. Thus, the neuroprotection by this extract is associated with the inhibition of oxidative stress signaling pathways.

### 2.3. The 75% EEPF Inhibits Glutamate-Induced Apoptotic Cell Death

**Figure 3:**
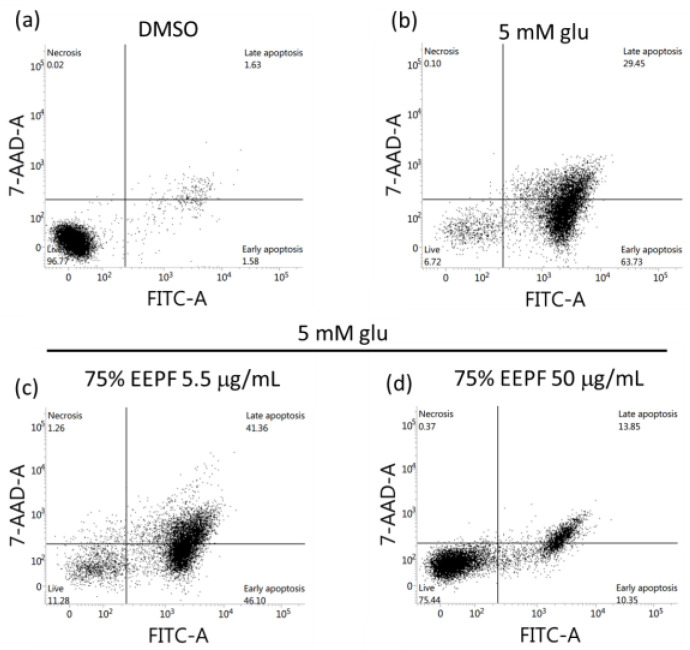
Apoptosis analysis by flow cytometry. HT22 cells were treated with glutamate and 75% EEPF for 18 h and flow cytometry was performed. (**a**) DMSO control shows 96.77% live cells. (**b**) 5 mM glutamate treated wells shows only 6.72% live cells and 93.18% cells has undergone apoptotic cell death. (**c**), (**d**) The 75% EEPF co-treatment has significantly suppressed the apoptosis at 50 µg/mL but failed suppress at low concentration 5.5 µg/mL.

According to the literature, glutamate-induced cell death is associated with apoptosis and necrosis in HT22 cells. Glutamate-treated cells undergo cell death mainly by apoptosis at early time points, and over time, cell death by necrosis is also involved [29]. However, 75% EEPF prevented glutamate-induced cell death. Therefore, we evaluated the association of these cell death phenotypes using annexin V and 7-AAD staining by flow cytometry. The flow cytometry results showed that cell death was primarily associated with apoptosis (7-AAD^−^/annexin V^+^ to 7-AAD^+^/annexin V^+^) and a small percentage of cell death by necrosis (7-AAD^+^/annexin V^−^). The 75% EEPF co-treatment prevented cell death irrespective of apoptosis and necrosis cell death (Figure 3). Glutamate treatment in HT22 cells increased sub-G1 fraction from 13.09% to 86.29%. However, the cotreatment of EEPF reduced sub-G1 fraction from 86.29% to 6.7%. This data indicated that EEPF could protect HT22 cells against oxidative stress induced apoptotic cell death (Figure S1).

**Figure 4:**
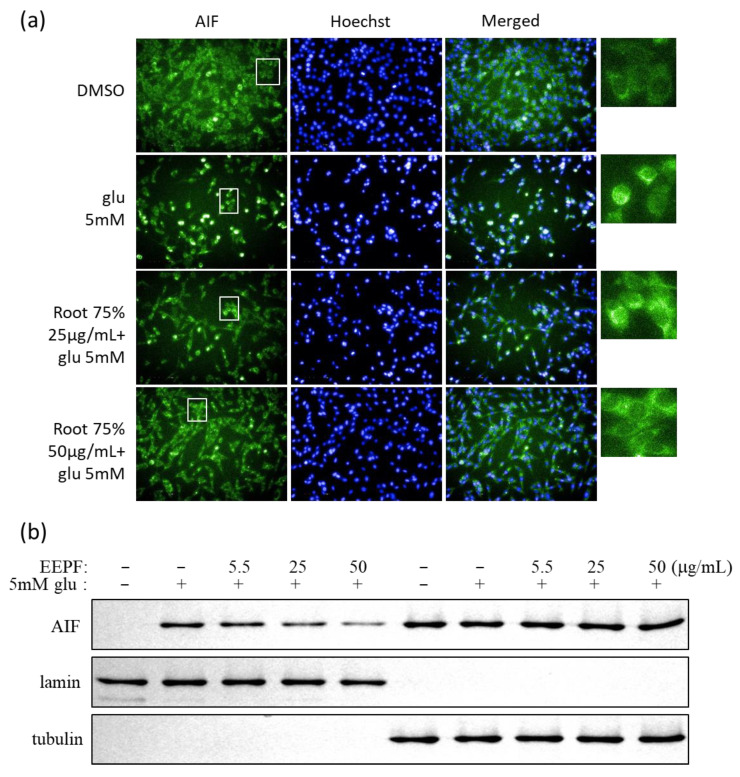

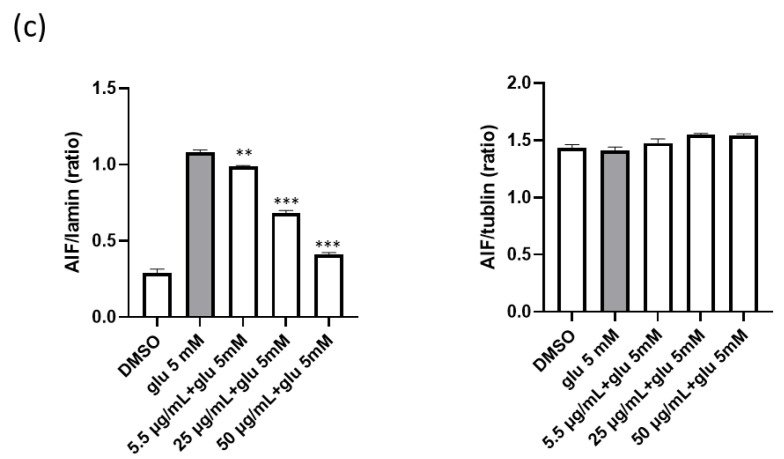
The 75% EEPF suppressed glutamate-induced AIF translocation to the nucleus. (**a**) Immunostaining of AIF and nucleus in HT22 cells after treatment of 5 mM glutamate and 75% EEPF (scale bar). (i) DMSO; (ii) An amount of 5 mM glutamate-induced AIF translocation into nucleus; (iii) An amount of 25 μg/mL; and (iv) An amount of 50 μg/mL of 75% EEPF has decreased translocation of AIF to the nucleus. (**b**) Nucleus and Cytosol was fractionationated and analyzed by western blot, the AIF was observed in nucleus fraction of glutamate and it gradually reduced with increasing concentrations of 75% EEPF; lamin (nucleus control); and tubulin (cytosol control). (**c**) Bar graph of the intensity of immunoblot bands visualized using the image J software (version 1.51j8). Data presented as the mean ± standard deviation (SD) of two independent experiments. *** *p* < 0.001, ** *p* < 0.01 vs. the glu 5 mM group.

Several studies have shown that glutamate-induced cell death is mediated by AIF [14]. Glutamate-mediated oxidative stress induces the release of mitochondrial AIF to the nucleus. Therefore, we evaluated whether EEPF inhibited AIF-mediated apoptotic cell death. Both immunostaining and western blot data showed that 75% EEPF prevented AIF translocation to the nucleus (Figure 4a,b). These data reveal that the 75% EEPF suppresses the apoptosis by inhibiting the AIF translocation.

### 2.4. The 75% EEPF Inhibits the MAPK and Activates AKT/BDNF/CREB Signal Pathway

**Figure 5:**
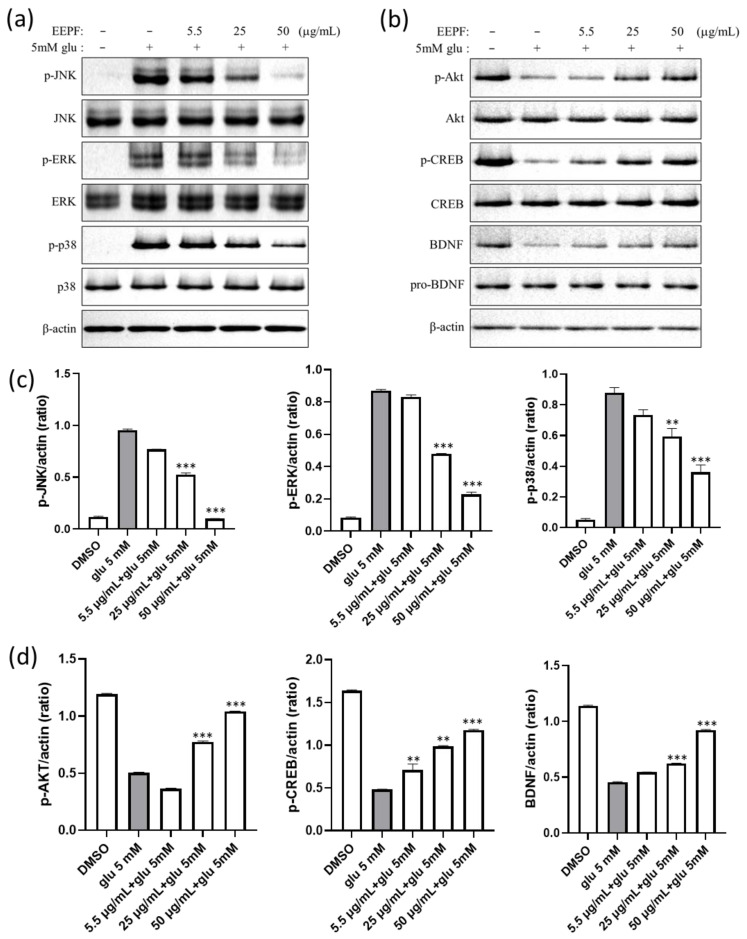
The 75% EEPF regulates MAPK activation and PI3K/AKT/CREB. (**a**) Protein expression levels of p-JNK, JNK, p-ERK, ERK, p-p38, and p38. (**b**) Protein expression levels of p-AKT, AKT, p-CREB, CREB, BDNF, and pro-BDNF. The glutamate and 75% EEPF was treated for 8 hrs and western blot was performed. (**a**) The 75% EEPF co-treatment inhibited p-JNK, p-ERK, and p-p38 protein level. (**b**) The 75% EEPF co-treatment rescued the p-AKT, p-CREB, and pCREB proteins levels. (**c**) and (**d**) Bar graphs of the intensity of immunoblot bands visualized using the image J software. Data presented as the mean ± standard deviation (SD) of two independent experiments. *** *p* < 0.001, ** *p* < 0.01 vs. the glu 5 mM group.

Glutamate also induced oxidative stress-sensitive MAPK pathway proteins, including p38, ERK1/2, and JNK. However, co-treatment with 75% EEPF inhibited the activation of MAPK (Figure 5a). Glutamate neurotoxicity suppresses the pro-survival pathway proteins PI3K/AKT and the transcription factor CREB. CREB regulates the expression of many neuroprotective genes, especially BDNF. The literature indicates that BDNF activation is associated with the activation of pro-survival Akt. To evaluate whether PF extracts activate the PI3K/AKT pathway, western blot analysis was performed for p-AKT, AKT, p-CREB, CREB, pro-BDNF, and BDNF. We found that treatment with 5 mM glutamate decreased p-AKT, p-CREB, and BDNF levels in HT22 cells. However, 75% EEPF co-treatment recovered these protein levels (Figure 5b) which indicates that the PF extract protects neuronal cells by activating the AKT/BDNF/CREB signaling pathway.

### 2.5. The 75% EEPF Reverses Glutamate-Mediated Altered Ratio of Bcl-2 and Bax Proteins

**Figure 6:**
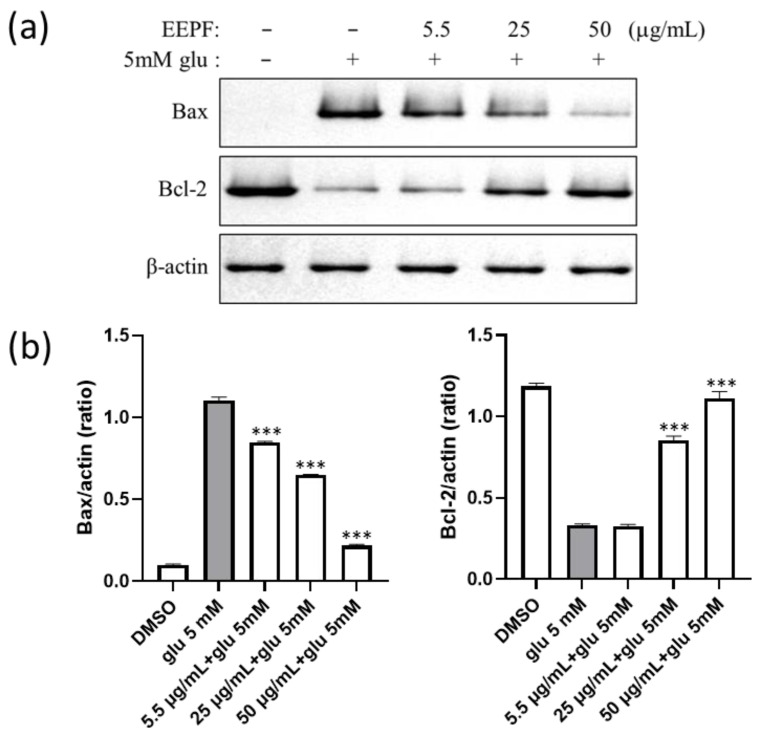
The 75% EEPF regulated apoptotic-related proteins. (**a**) Protein expression levels of Bax and Bcl-2. The 75% EEPF co-treatment decreased Bax protein and increased the Bcl-2 proteins levels and prevented the cell death. (**b**) Bar graph of the intensity of immunoblot bands visualized using the image J software. Data presented as the mean ± standard deviation (SD) of two independent experiments. *** *p* < 0.001 vs. the glu 5 mM group.

The Bcl-2 protein family regulates pro- and anti-apoptotic factors and is associated with glutamate-induced cell death. Glutamate changes the anti-apoptotic Bcl-2 and pro-apoptotic Bax ratio, which selectively regulates both pro-survival and cell death pathways. Therefore, we analyzed these two protein levels after 75% EEPF treatment in HT22 cells by immunoblot. The results demonstrated that glutamate increased Bax and decreased Bcl-2 levels. However, *Polyscias* treatment reduced Bax and increased Bcl-2 levels, which prevented the activation of apoptotic cell death mediated by glutamate (Figure 6a).

### 2.6. Neuroprotective Effects of 75% EEPF against Ischemic Brain Injury

**Figure 7:**
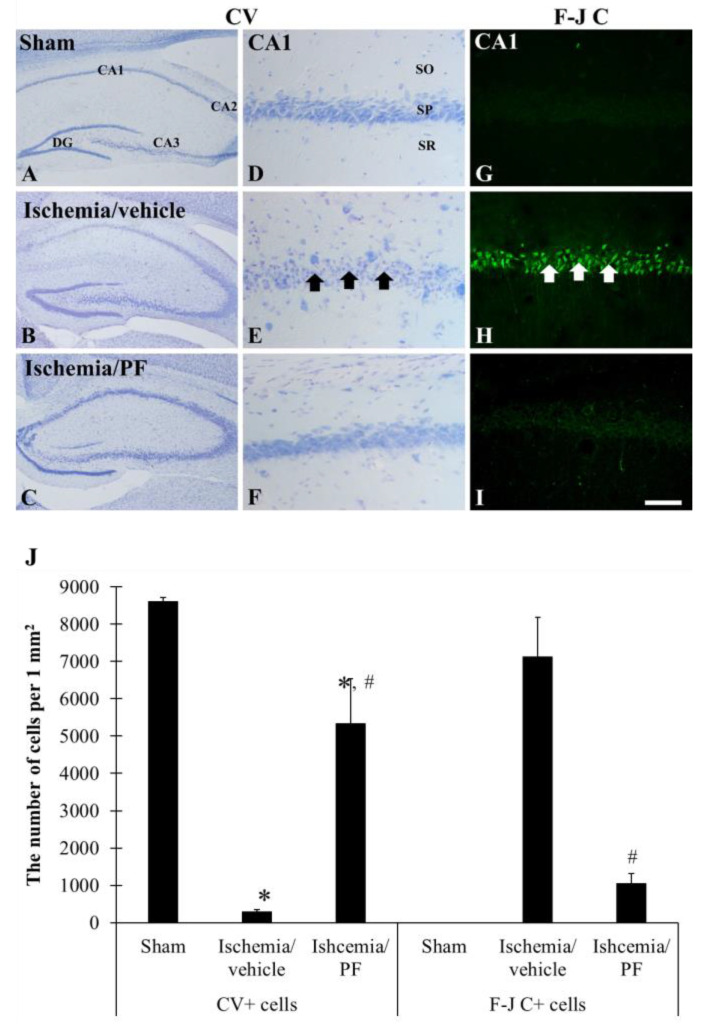
The 75% EEPF neuroprotection of ischemic neuronal damage. Cresyl violet (CV) (**A**–**F**) and Fluoro-Jade C (F-J C) (**G**–**I**) staining in the hippocampus (**A**–**C**) and Cornu Ammonis 1 (CA1) region (**D**–**I**) of the sham (**A**,**D**,**G**), ischemia/vehicle (**B**,**E**,**H**), ischemia/PF (**C**,**F**,**I**) groups at 5 days after ischemic surgery. In the ischemia/vehicle groups, CV positive (+) cells are significantly reduced (black arrows) and many Fluoro-Jade C+ cells are observed (white arrows) in the stratum pyramidale within the CA1 region. In the ischemia/PF group, CV+ and F-J C+ cell density is close to that in the sham group. Scale bar = 400 μm (**A**–**C**), 100 μm (**G**–**I**,**J**). Number of CV+ and F-J C+ cells in the CA1 region is n = 7 for each group. * *p* < 0.05 versus sham group, # *p* < 0.05 versus ischemia/vehicle group. The bars indicate the means ± SEM.

We further investigated the neuroprotective efficacy of 75% EEPF in Mongolian gerbils with induced ischemic brain injury. The gerbils were treated daily with 75% EEPF (20 mg/kg body weight) for three days. Cerebral ischemia was transiently induced in the gerbil brain by surgery. The brain tissue sections were analyzed by histopathology with CV and F-J C staining to study the normal and the degenerated neurons, respectively (Figure 7). The control group showed the highest number of CV+ cells in the pyramidal striatum of the hippocampus. However, the ischemia-induced group showed a significant reduction in the CV+ stains. The 75% EEPF treatment group showed that the CV+ cells were similar to the control group, indicating a strong neuroprotective effect from ischemic damage. The F-J C staining data showed a significant increase in F-J C+ neurons in the ischemia-induced group compared with the control group (Figure 7G–I). However, the 75% EEPF treated group showed a dramatic reduction in F-J C+ stained neurons, indicating significant neuroprotection from ischemic damage (Figure 7). These results support the neuroprotective effect of 75% EEPF in in vivo models.

We also investigated whether 75% EEPF blocks ischemia-induced glial activation. We performed immunostaining of glial fibrillary acidic proteins (GFAP) in astrocytes obtained from Mongolian gerbils. GFAP is a cytoskeletal marker in mature astrocytes. GFAP immunoreactivity was increased in response to injury to the central nervous system [30]. In the sham group, GFAP immunoreactive astrocytes showed characteristics of a resting form that possessed small cytoplasm and thread-like processes. In the ischemia group, GFAP immunoreactive astrocytes showed an active form that possessed hypertrophic cytoplasm. The morphology of GFAP immunoreactive astrocytes in the 75% EEPF-treated group was similar to that of the sham group.

**Figure 8:**
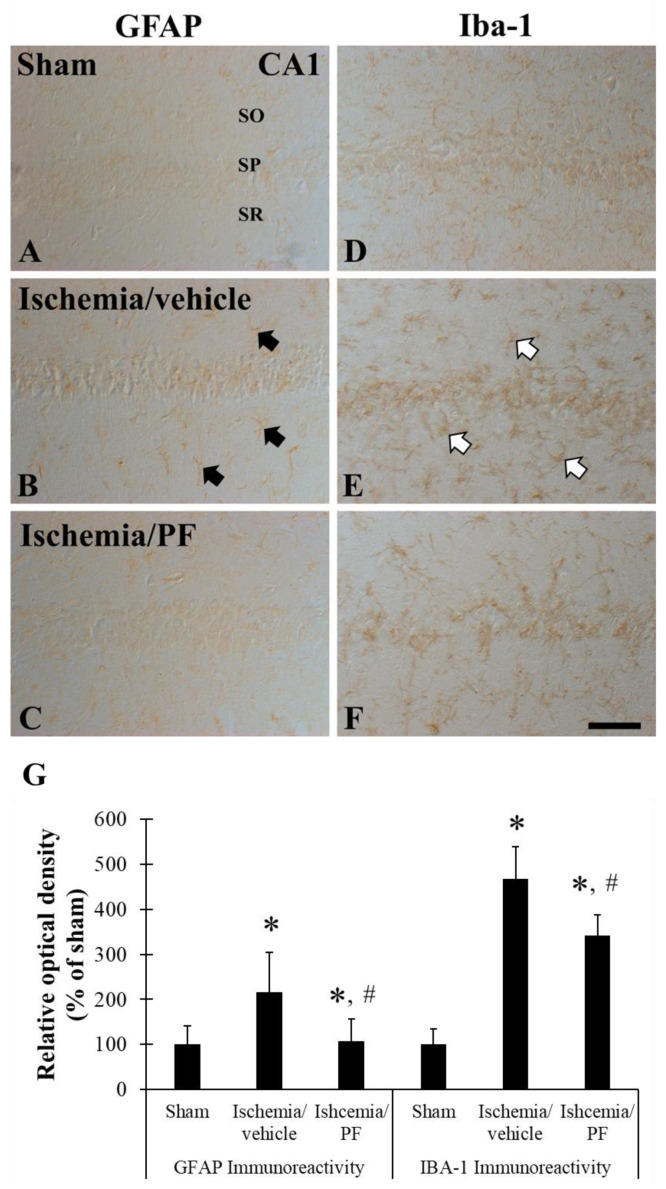
The 75% EEPF suppresses the ischemia-induced glial cell activation. Glial fibrillary acidic protein (GFAP) (**A**–**C**) Ionized calcium binding adaptor molecule-1 (Iba-1) (**D**–**F**) immunohistochemistry in the hippocampal CA1 region (**D**–**I**) of the sham (**A**,**D**), ischemia/vehicle (**B**,**E**), ischemia/PF (**C**,**F**) groups at 5 days after ischemic surgery. In the ischemia/vehicle groups, GFAP (black arrows) and Iba-1 (white arrows) immunoreactivities are increased in the CA1 region. In the ischemia/PF group, GFAP and Iba-1 immunoreactivities are decreased compared with that in the ischemia/vehicle group. Scale bar = 100 μm (**G**). Relative optical density (ROD) of GFAP and Iba-1 in the CA1 region (n = 7 for each group, * *p* < 0.05 versus sham group, # *p* < 0.05 versus ischemia/vehicle group). The bars indicate the means ± SEM.

In the lba-1 immunostaining of microglia, the immunostaining of microglia in the sham group showed a typical resting form of microglia that possess small cytoplasm and thread-like processes. Under ischemic conditions, Iba-1 immunoreactive microglia showed reactive glial forms that had hypertrophic cytoplasm and retracted processes. In particular, many Iba-1 immunoreactive reactive microglia were observed in the stratum pyramidal (SP) layer of the hippocampal CA1 region where neuronal cell death occurred. Treatment with 75% EEPF decreased the number of reactive microglia compared with ischemic conditions. In particular, 75% EEPF treatment resulted in a prominent reduction in Iba-1 immunoreactive reactive microglia in the SP layer. The relative optical density (ROD) for Iba-1 immunoreactivity showed the same results (Figure 8). These data firmly support the neuroprotective effect of 75% EEPF in induced ischemic brain injury in in vivo models.

### 2.7. Identification of Novel Compounds in EEPF

**Figure 9:**
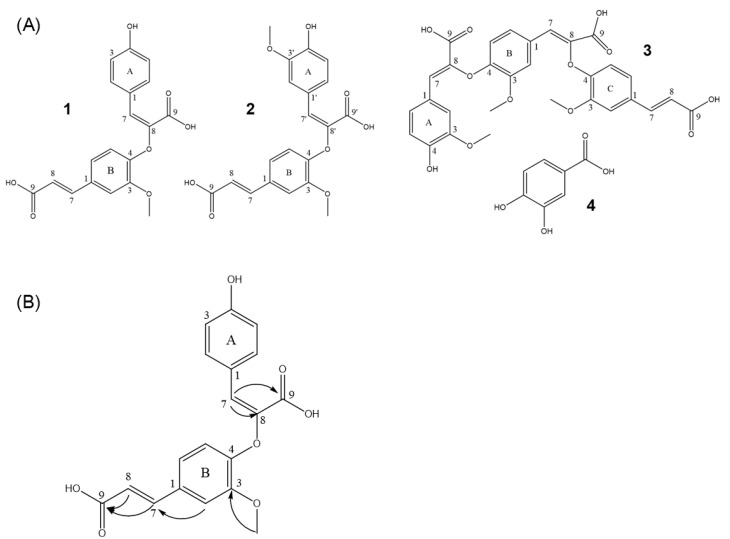
(**A**) Structures of isolated compounds (**1**–**4**). (**B**) Key HMBC correlations for compound **1** (from proton to carbon).

Repeated column chromatography of an EtOAc-soluble led to the isolation of a new compound, politoic acid (1), along with three other known compounds (2–4). From analysis of the reported physical and spectroscopic data, the chemical structures of known compounds were identified as 8-O-4-dehydrodiferulic acid (2) [31], 8-O-4/8-O-4-dehydrotriferulic acid (3) [32], and protocatechuic acid (4) [33,34] (Figure 9A).

Compound 1 was obtained as a yellowish gum with a molecular formula of C_19_H_16_O_7_ from the [M+Na]^+^ peak at m/z 379.0793 [M+Na]^+^ calculated for C_19_H_16_O_7_Na 379.0794 in positive-ion HRESI-MS (Figure S3). Using 1D and 2D NMR experiments (^1^H, ^13^C, HSQC, HMBC), compound 1 was unambiguously identified as politoic acid (Figure 9 and Figure S3–S7). The proton spectrum showed the signals of three protons belonging to one methoxy group, and the position of carbon 3A was determined by HMBC (Figure S8). The aromatic region (δ7.33 [1H, s], 6.79 [1H, d, *J* = 8.25], 7.08 [1H, d, *J* = 8.30] and 7.57 [2H, d, *J* = 8.45], 6.74 [2H, d, *J* = 8.50]) indicated the presence of 1,3,4-trisubstituted and 1,4-disubstituted benzene rings. Two 15.9 Hz doublets in the proton spectrum revealed an unsubstituted trans-cinnamic acid side chain, and a singlet at 7.37 ppm characteristic of an unsaturated 7-proton suggested one coupling via the 8-position. The two quaternary carbons 9A and 8A in the 1D carbon spectrum were too weak to be deduced. These carbon shifts were observed in the 2D HMBC spectra (Figure S8). All correlation signals in the HMBC aligned fully with the structure shown in Figure 9B. Moreover, the NMR data for 1 were similar to those for 2, except for the absence of a methoxy group signal that led to the difference in the benzene ring signals (Table 1). Therefore, the structure of 1 was identified as (E)-2-(4-((E)-2-carboxyvinyl)-2-methoxyphenoxy)-3-(4-hydroxyphenyl)acrylic acid, and it was named politoic acid. We next examined whether these compounds showed neuroprotective effects against glutamate-induced neuronal cell death. The compounds 2 and 3 showed slight neuroprotective effects at the range of 3.7 μM to 11.1 μM (Figure S2). These data suggest that compounds 2 and 3 could be the active constituents of EEFP for neuroprotection in HT22 cells.

## 3. Discussion

*P. fruticosa* has been traditionally used as an herbal medicine for the treatment of ischemia, inflammation, and various health ailments. However, to date, no studies have been reported regarding the neuroprotective effects of these plant extracts against glutamate-induced cell death and ischemic brain injury in Mongolian gerbil models. Abnormal glutamate levels alter intracellular oxidative stress by affecting intracellular Ca^2+^ levels, ROS, and mitochondrial dysfunction, leading to the activation of apoptotic cell death. Therefore, targeting the glutamate-induced cell death pathway has become a viable therapeutic approach.

Here, for the first time, we demonstrated the neuroprotective effect of *P. fruticosa* extracts in HT22 cells. The 75% EEPF protected HT22 cells from glutamate-induced cell death and reduced ROS and Ca^2+^ influx accumulation. *P. fruticosa* root extract prevented glutamate-induced neuronal toxicity by blocking AIF translocation from the mitochondria to the nucleus and activating the AKT/BDNF/CREB pathway. Moreover, 75% EEPF inhibited the activation of the anti-apoptotic protein Bcl-2 and activated the downregulation of the pro-apoptotic protein Bax. Additionally, 75% EEPF induced CREB, which led to the activation of BDNF and PI3K/AKT pathways and suppressed the activation of MAPK pathway proteins induced by high glutamate levels. MAPK proteins are sensitive to oxidative stress and are involved in the activation of apoptotic cell death.

Furthermore, the PF root-75% EEPF neuroprotection was studied in ischemic brain injury in in vivo models. Ischemic brain injury was induced in Mongolian gerbils after treatment with 75% EEPF. The 75% EEPF significantly reduced the generation of degenerated neurons in the CA1 region of the hippocampus. Further, the EEPF prevented the activation of glial cells, the immunoreactive astrocytes were not active, and it was similar to the sham group. It is well known that ischemia induces microglia activation [35]. The activated microglia cause neuroinflammatory response by releasing neurotoxic product and proinflammatory cytokines, which causes neuronal cell death. The suppression of microglia activation by EEPF can attenuate cerebral ischemic damage [36].

According to the literature, AKT/BDNF/CREB activation inhibits the adverse effects of glutamate and ischemic brain damage [27]. The activation of AKT/CREB and the down-regulation of the MAPK pathway protects the neurons from cell death and prevents neuronal loss in Mongolian gerbil brain ischemia [37,38]. Similarly, the EEPF treatment in HT22 followed this pattern and prevented glutamate neurotoxicity. Therefore, we assume that the EEPF neuroprotection in Mongolian gerbils was strongly associated with the up and down-regulation of AKT and MAPK, respectively.

Our study demonstrates the neuroprotective effects and underlying molecular mechanisms of *P. fruticosa* under glutamate-triggered cell death. The findings of this study demonstrate the therapeutic potential of *P. fruticosa* against glutamate neurotoxicity and ischemic brain injury.

## 4. Materials and Methods

### 4.1. General Experimental Procedures

A 500 MHz Bruker Avance DRX spectrometer was used to obtain the 1D-NMR and 2D-NMR spectra, and the δ values (ppm) were used for chemical shifts. A high-resolution electrospray ionization mass spectrometer was used to record the mass spectra. Column chromatography was carried out with silica gel (63–200 μm), RP-18 (75 μm), and TLC with RP-18 F254 plates and silica gel 60. A 10% H_2_SO_4_ solution was used to visualize the isolated compounds, followed by heating for 5 min. Open column chromatography was conducted using silica gel (Merck Corp., Darmstadt, HE, Germany) and Sephadex LH-20 ((Merck Corp., Darmstadt, HE, Germany).

### 4.2. Preparation of EEPF (Ethanol Extract of Polyscias fruticosa)

*P. fruticosa* was purchased from Vietnam in June 2018 and identified by Dr. Thi Thu Thuy Tran Institute of Natural Products (INPC), Vietnamese Academy of Science and Technology (VAST), Vietnam. The plants were separated into roots. The samples were dried at room temperature for a week and ground to a fine powder. In total, 5 g of each sample, including the roots and leaves, were extracted with 100 mL of different percent of ethanol solution (EtOH 95%, 75%, 50%, 25%, and 0%) and water twice for 3 h via reflux.

After the extraction, the following quantities of extracts were obtained for each plant part. From 95% EtOH: Roots, 824.2 mg (16.5%). From 75% EtOH: Roots, 2.92 g (58.4%). From 50% EtOH: Roots, 1.26 g (25.1%). From 25% EtOH: Roots, 1.24 g (24.7%). From 0% EtOH: Roots, 916.5 mg (18.3%).

### 4.3. Extraction and Isolation

Distilled water was used to dissolve the isolated ethanol extract, which was continuously separated using ethyl acetate (EtOAc) and n-butanol (Bu). The soluble fraction of EtOAc was loaded onto a separation column under a stepwise gradient of n-hexane-EtOAc (10:1 to 0:1) to yield five fractions (Fr.1-Fr.5) according to the TLC profiles. Then, Sephadex LH-20 open column was used to add fraction 1 and was eluted with MeOH-H2O (1-1) to yield compound 4 (3.2 mg). Fraction 2 was purified using a Sephadex LH-20 open column and eluted with MeOH to yield compound 3 (22.4 mg). Fraction 3 was chromatographed on a Sephadex LH-20 column eluted with MeOH-H_2_O (7-3) to yield compounds 1 (2.0 mg) and 2 (3.5 mg). Politoic acid: yellowish gum; ^1^H-NMR and ^13^C-NMR (methanol-*d*_4_) data, see Table 1; HRESI-MS m/z: 379.0793 [M+Na]^+^(calcd. for C_19_H_16_O_7_Na 379.0794).

### 4.4. Cell Culture

The HT22 cell line was obtained from the Korean Cell Line Bank (Seoul, South Korea). The cells were grown in DMEM-high glucose media (Hyclone laboratories Inc., Logan, UT, USA) supplemented with 10% fetal bovine serum (Gibco Co. Waltham, MA, USA), 1% penicillin/streptomycin, and 4 mM L-glutamine (Invitrogen, Waltham, MA, USA) at 5% CO_2_ and 37 °C.

### 4.5. Calcein-AM Live-Cell Assay

Calcein-AM (Invitrogen, Waltham, MA, USA) is a live cell-specific fluorescent dye. It diffuses into live cells, is hydrolyzed by active intracellular esterases, and emits green fluorescence. HT22 cells (3000 cells/well) were seeded into a 96-well plate. After overnight growth, the cells were treated with extracts, and 2 h later, 5 mM glutamate was added. After 12 h incubation, cell death reached approximately 50% in the glutamate-treated wells. Then, calcein-AM 1 µM solution prepared in DPBS was added to each well. Live cells were imaged using the operetta high throughput image system (Perkin Elmer, Waltham, MA, USA) using the GFP channel (ex/em, 488 nm/514 ± 15), and the live cells were counted using the Harmony 3.5 software (Perkin Elmer, Waltham, MA, USA).

### 4.6. DCF-DA ROS Assay

DCF-DA is a ROS-sensitive dye used to measure intracellular ROS. During the experiment, HT22 cells (3000 cells/well) were seeded in 96 well plates and grown overnight. The extracts and glutamate were treated for 8 h. Then cells were washed with serum-free media and 100 µL of 5 µM DCF-DA solution prepared in serum-free media was added. After 15 min incubation at 37 °C, the dye solution was removed and washed with serum-free media. Then, cells were imaged with the operetta high throughput image system (Perkin Elmer, USA) using the GFP channel (ex/em, 488 nm/514 ± 15). Fluorescence intensity was calculated using Harmony 3.5 (Perkin Elmer, Waltham, MA, USA) software.

### 4.7. Fluo-3 AM Calcium Assay

Fluo-3 AM is a calcium-sensitive dye; Ca^2+^ binding enhances the fluorescence of fluo-3 and is used to measure intracellular calcium levels. After glutamate treatment for 8 h, the medium was removed and washed with HBSS buffer (Cat No; 14025092, Thermo Fischer Scientific, Waltham, MA, USA). Fluo-3, 2 µM solution prepared in HBSS buffer was added and incubated for 15 min at 37 °C, 5% CO2. After incubation, cells were washed with HBSS buffer, and images were taken using the operetta high throughput image system (Perkin Elmer, Waltham, MA, USA) using the GFP channel (ex/em, 488 nm/514 ± 15). The fluorescence intensity of images were calculated using Harmony 3.5 (Perkin Elmer, Waltham, MA, USA) software.

### 4.8. Western Blot Analysis

The 1 × 10^6^ cells were seeded per well in a 6-well cell culture plate (Corning, Somerville, MA, USA). After overnight growth, the cells were treated with extracts and glutamate for 8 h. Then, cell lysis was prepared using cell extraction buffer (Invitrogen, Waltham, MA, USA) with a complete protease inhibitor cocktail (Sigma-Aldrich, St. Louis, MI, USA), and PMSF (1 mM). Cells were lysed for 30 min and centrifuged at 14,000 rpm for 20 min. Total protein content in the supernatant was quantified using a BCA assay kit (Invitrogen, Waltham, MA, USA). Then, 10 µg of protein was loaded onto an SDS-PAGE gel and separated at 100 V. The proteins on the gel were transferred to a PVDF membrane (Millipore, Burlington, MA, USA) at 100 V for 90 min. Subsequently, blocking was performed using 5% skimmed milk or 3% BSA (for phosphorylated proteins) for 1 h. Then, the primary antibody (1:1000 dilution) treatment was performed overnight at 4 °C. Then, the secondary antibodies (1:1000 dilution) were incubated at room temperature for 90 min. The chemiluminescent images were taken using a super-signal West Pico Plus reagent (Thermo Fisher Scientific, Waltham, MA, USA) and image analyzer LAS-3000 (Fujifilm, Minato City, Tokyo, Japan) document system.

### 4.9. Apoptosis Analysis by Flow Cytometry

The HT22 4 × 10^5^ cells were seeded in a 100 mm culture dish. After overnight growth, the cells were treated with 75% *P. fruticosa* (PF) root extract and 5 mM glutamate. After 18 hrs, the cells were harvested and washed with DPBS. Then, cells were mixed with 1X annexin V buffer and 1 × 10^5^ cells were transferred to a separate tube. Then, 10 µL of annexin V and 5 µL of 7-AAD dyes were added. After 15 min incubation, the flow cytometry was performed using BD FACS Verse (BD Biosciences, Franklin Lakes, NJ, USA).

### 4.10. Pretreatment of EEPF In Vivo Study

Male Mongolian gerbils (age 6 weeks, 60–70 g) were used for an ischemic brain injury model. The protocol was approved by the Animal Committee at Gangneung Wonju National University. The Mongolian gerbils were categorized as follows (n = 7 per group): (1) sham, (2) vehicle-treated ischemic gerbils, and (3) EEPF-treated ischemia gerbils. EEPF (75%) was mixed in the vehicle (saline:ethanol:Tween 80 = 8:1:1) and treated to Mongolian gerbils.

### 4.11. Induction of Transient Cerebral Ischemia

For the operation of Mongolian gerbils, we used 2.5% isoflurane (Bzxtor healthcare corporation, Deerfield, IL, USA) in 32.2% oxygen and 65.3% nitrogen. After anesthesia, carotid artery obstruction in Mongolian gerbils was performed using clips for 5 min. A rectal temperature probe and thermometer blanket (TR-100; Fine Science Tools, Heidelberg, Germany) were utilized to maintain the body temperature (37 ± 0.5 °C) during the operation. After the operation, the Mongolian gerbils were recovered in the incubator. The carotid arteries of the sham animals were unblocked. However, the other procedures were performed in the same way.

### 4.12. Histology

Five days after surgery for ischemic induction, the Mongolian gerbils (n = 7 per group) were injected with sodium pentobarbital (30 mg/kg, i.p.) in 0.1 M PBS (pH 7.4). Mongolian gerbils were injected transcardially with 4% paraformaldehyde in 0.1 M phosphate buffer (pH 7.4). The brain samples were fixed with 4% paraformaldehyde for 6 h. For cryoprotection, the fixed brain tissue samples were infiltrated with 30% sucrose overnight. The brain tissue samples were cut to 30 μm thickness using a cryostat (Leica, Germany) for further analysis.

### 4.13. Cresyl Violet Staining

The gelatin-coated glass slides were used for the attachment of brain slices. The tissue slides were treated with 10% cresyl violet solution for 30 min. Then, washed with tap water and dehydrated in serial ethanol jars. The dehydrated brain tissue was sealed with permount (Sigma, USA).

### 4.14. Staining with Fluoro-Jade C

The tissue slides were incubated with 1% sodium hydroxide solution and 80% ethanol for 5 min and dehydrated in 70% ethanol-distilled water. Then, slides were treated with 0.06% KMnO_4_ solution for 10 min, followed by 0.0001% fluoro-Jade C for 30 min. The tissue samples were washed with distilled water and dried in a dry oven. The tissue samples were sealed with DPX (Sigma, USA) and images were taken using an epifluorescence microscope (Axio M1, Carl Zeiss, Gottingen, Germany) at 450–490 nm.

### 4.15. Statistical Analysis

Statistical analysis and bar graphs were generated using GraphPad prism 6.0 (San Diego, CA, USA). The statistical significance of the compound treatment groups were analyzed by one-way analysis of variance (ANOVA). For tissue image analysis, the statistical difference between the groups were analyzed by one-way ANOVA followed by Duncan’s post hoc test using SPSS software (SPSS ver 17.0, Inc., Chicago, IL, USA).

## 5. Conclusions

In this study, we have found the neuroprotective effects of EEPF (ethanol extract *Polyscias fruticosa*) against glutamate-induced neuronal cell death in HT22 cells. EEPF exhibited its neuroprotective effects via an inhibition of the MAPK and the activation of the AKT/BDNF/CREB signal pathway in glutamate-treated HT22 cells. Furthermore, EEPF blocked glutamate-induced AIF translocation from mitochondria to the nucleus. EEPF showed neuroprotective effects in an ischemia model using Mongolian gerbils. These results revealed that EEPF may provide a potential therapeutic application for brain ischemia.

## Figures and Tables

**Table 1:** NMR data for compounds **1** and **2**.

		^1^H (Methanol-*d*_4_, 500 MHz)		^13^C(Methanol-*d*_4_, 125 MHz)	
		1	2	1	2
benzene ring A	1			126.5	126.9
	2	7.57 (1H, d, *J* = 8.45)	7.44 (1H, s)	134.5	115.0
	3	6.74 (1H, d, *J* = 8.45)		117.9	150.2
	4			161.6	151.1
	5	6.74 (1H, d, *J* = 8.50)	6.76 (1H, d, *J* = 8.25)	117.9	117.5
	6	7.57 (1H, d, *J* = 8.45)	7.11 (1H, d, *J* = 8.30)	134.5	127.7
	7	7.37 (1H, s)	7.41 (1H, s)	129.8	130.5
	8			140.5	140.0
	9			168.2	168.0
	OCH_3_		3.70 (3H, s)		57.2
benzene ring B	1			118.8	119.0
	2	7.33 (1H, s)	7.33 (1H, s)	114.1	113.9
	3			151.9	151.8
	4			150.7	150.5
	5	6.79 (1H, d, *J* = 8.25)	6.79 (1H, d, *J* = 8.40)	116.3	115.9
	6	7.08 (1H, d, *J* = 8.30)	7.08 (1H, d, *J* = 8.40)	124.4	124.4
	7	7.62 (1H, d, *J* = 15.9)	7.62 (1H, d, *J* = 15.9)	147.3	147.2
	8	6.39 (1H, d, *J* = 15.9)	6.40 (1H, d, *J* = 15.9)	118.9	118.9
	9			171.9	171.8
	OCH_3_	3.99 (3H, s)	3.99 (3H, s)	58.0	58.0

## Supplementary Materials

The following supporting information can be downloaded at: https://www.mdpi.com/article/10.3390/ijms24043969/s1.
