# Supplementary material for: Neuroprotective Effects of Ethanol Extract of Polyscias fruticosa (EEPF) against Glutamate-Mediated Neuronal Toxicity in HT22 Cells
Source: Int J Mol Sci. 2023 Feb 16;24(4):3969. doi: 10.3390/ijms24043969 (PMC9959701; doi:10.3390/ijms24043969)
Supplement: Supplementary file 1 [file ijms-24-03969-s001.zip › ijms-2185647-supplementary-updated.pdf]

Neuroprotective effects of ethanol extract of *Polyscias fruticosa* (EEPF) against glutamate-mediated neuronal toxicity in HT22 cells

Baskar Selvaraj[1,8], Tam Tie Le[1,8,9], Dae Won Kim[2], Bo Hyun Jung[3], Ki Yeun Yoo[3], Hong Ryul Ahn[1], Phuong Thien Thong[4], Thi Thu Thuy Tran[5,6], Ae Nim Pae[7], Sang Hoon Jung[1,8,*], Jae Wook Lee[1,8,*]

[1]Natural Product Research Center, Institute of Natural Product, Korea Institute of Science and technology, Gangneung, 25451, Republic of Korea.

[2]Department of Biochemistry and Molecular Biology, Research Institute of Oral Science, College of Dentistry, Gangneung Wonju National University, Gangneung, 25457, Republic of Korea.

[3]Department of Anatomy, College of Dentistry, Gangneung Wonju National University, Gangneung, 25457, Republic of Korea.

[4]Devision of Biotechnology, Vietnam Korea Institute of Science and Technology, Hoa lac High-tech Park, km29 Thang Long Boulevard, Hanoi, Vietnam

[5]Institute of Natural Products Chemistry, Vietnam Academy of Science and Technology (VAST), 18 Hoang Quoc Viet, Caugiay, Hanoi, Vietnam

[6]Department of Chemistry, Graduate University of Science and Technology, VAST, 18 Hoang Quoc Viet, Caugiay, Hanoi, Vietnam

[7]Center of Brain Disorders, Brain Science Institute, Korea Institute of Science and Technology, Seoul, 02792, Republic of Korea

[8]Division of Bio-medical Science & Technology, University of Science and Technology, Daejun, 34113, Republic of Korea.

[9]These authors equally contributed in this work

* Correspondence: shjung@kist.re.kr; Tel.: (82-33-650-3653, S. H. Jung) jwlee5@kist.re.kr; Tel.:(82-33-650-3514, J.W. Lee)

List of Figures

Figure S1. Cell cycle analysis of apoptotic cells using PI. .

Figure S2. Protective effects of novel compounds in EEPF.

Figure S3. EIMS spectrum of new compound **1**

Figure S4. ^{1}H-NMR spectrum of new compound **1** in CH_3OH-d_4 at 500 MHz

Figure S5. ^{13}C-NMR spectrum of new compound **1** in CH_3OH-d_4 at 125 MHz

Figure S6. HSQC spectrum of new compound **1** in CH_3OH-d_4

Figure S7. COSY spectrum of new compound **1** in CH_3OH-d_4

Figure S8. HMBC spectrum of new compound **1** in CH_3OH-d_4

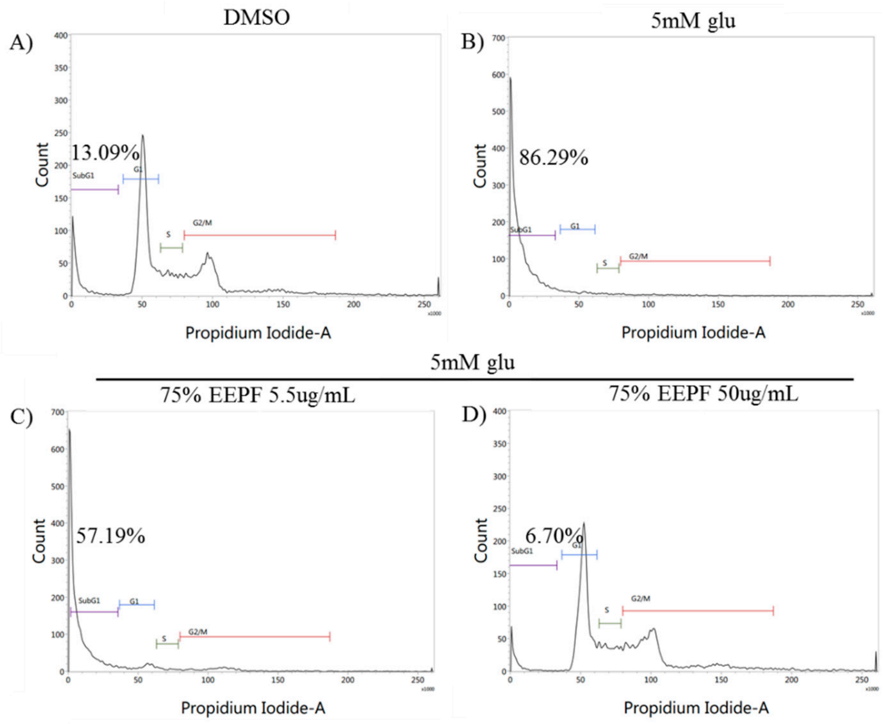

Figure S1. DNA contents of apoptotic cells in sub-G1 phase was determined by flow cytometry using PI (propidium iodide). The 5 mM glutamate has increased sub-G1 phase to 86.29%. However, the cotreatment of 50 μg/ml of EEPF restored ratio of sub-G1 phase to 6.70%.

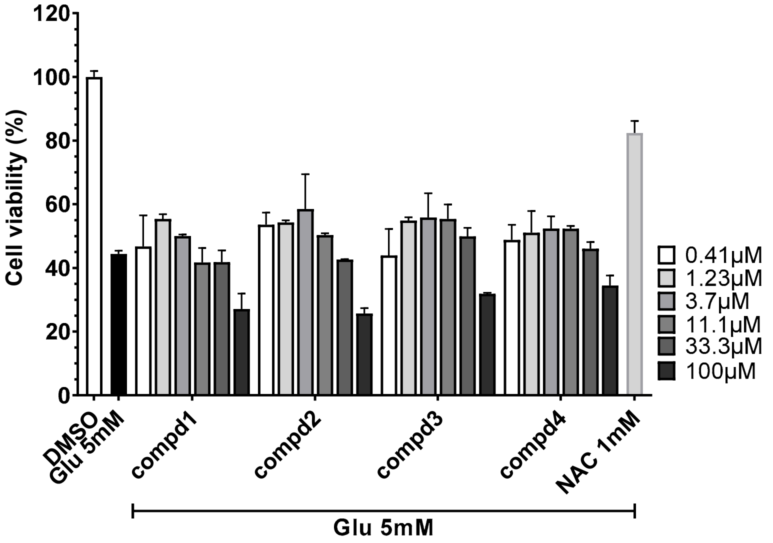

Figure S2. Protective effects of novel compounds in EEPF. The HT22 cells were treated with compounds, and 2 hr after 5 mM glutamate was treated and after 12 hrs the cell viability was measured by EZ-Cytox reagent. Data bar graphs are presented as means ± SD, n=3.

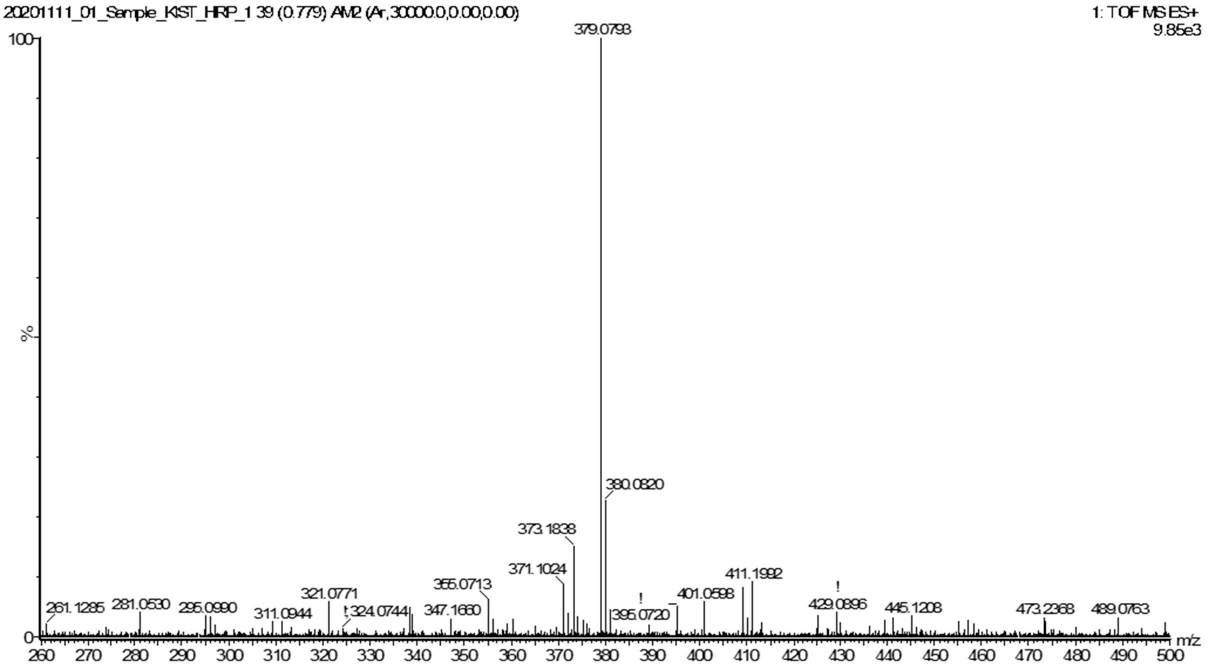

Figure S3. HRESI-MS spectrum of new compound **1**

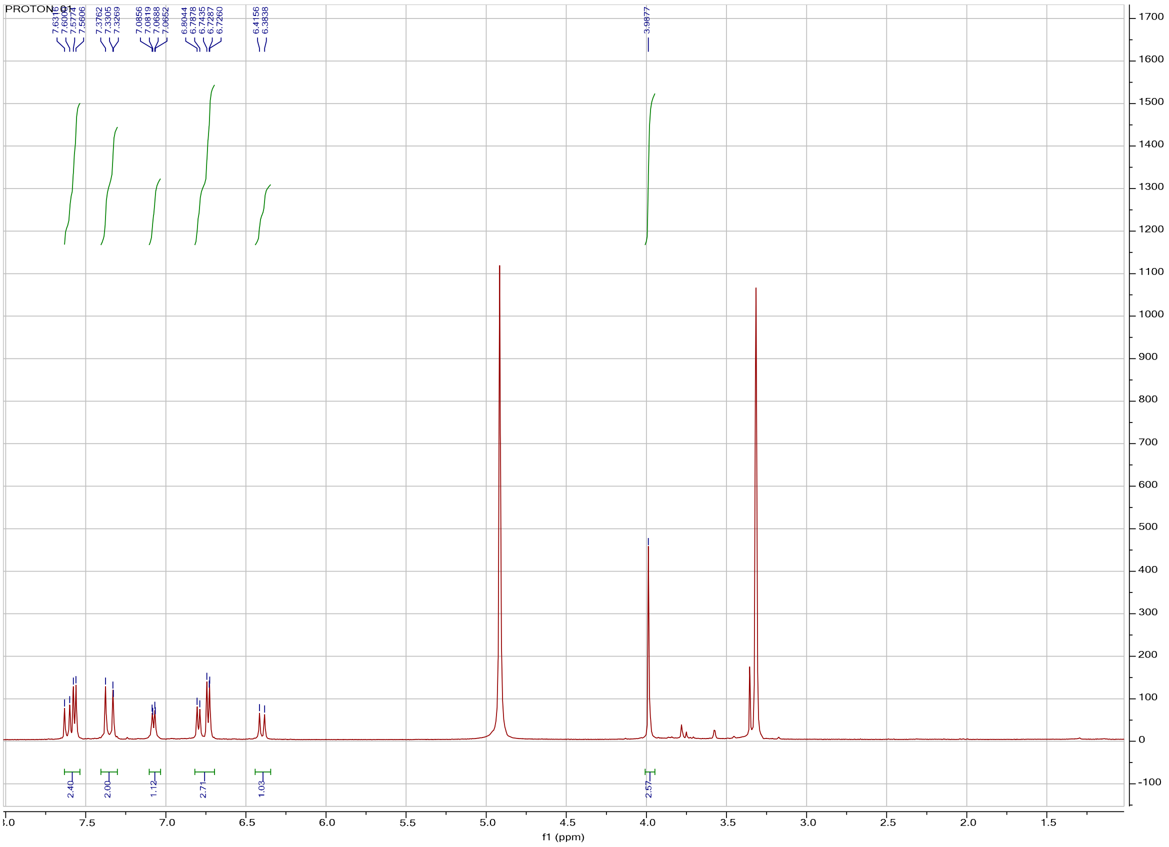

Figure S4. ^{1}H-NMR spectrum of new compound 1 in CH_3OH-d_4 at 500 MHz

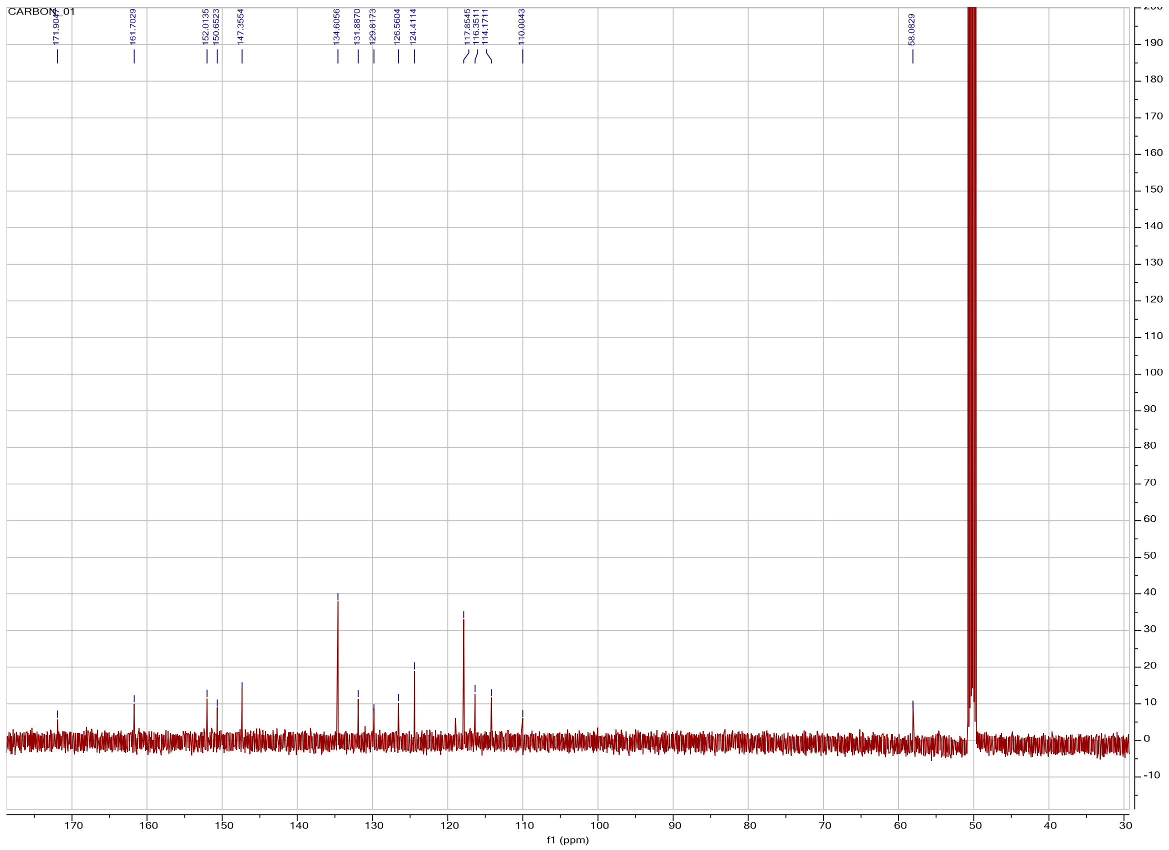

Figure S5. ^{13}C-NMR spectrum of new compound 1 in CH_3OH-d_4 at 125 MHz

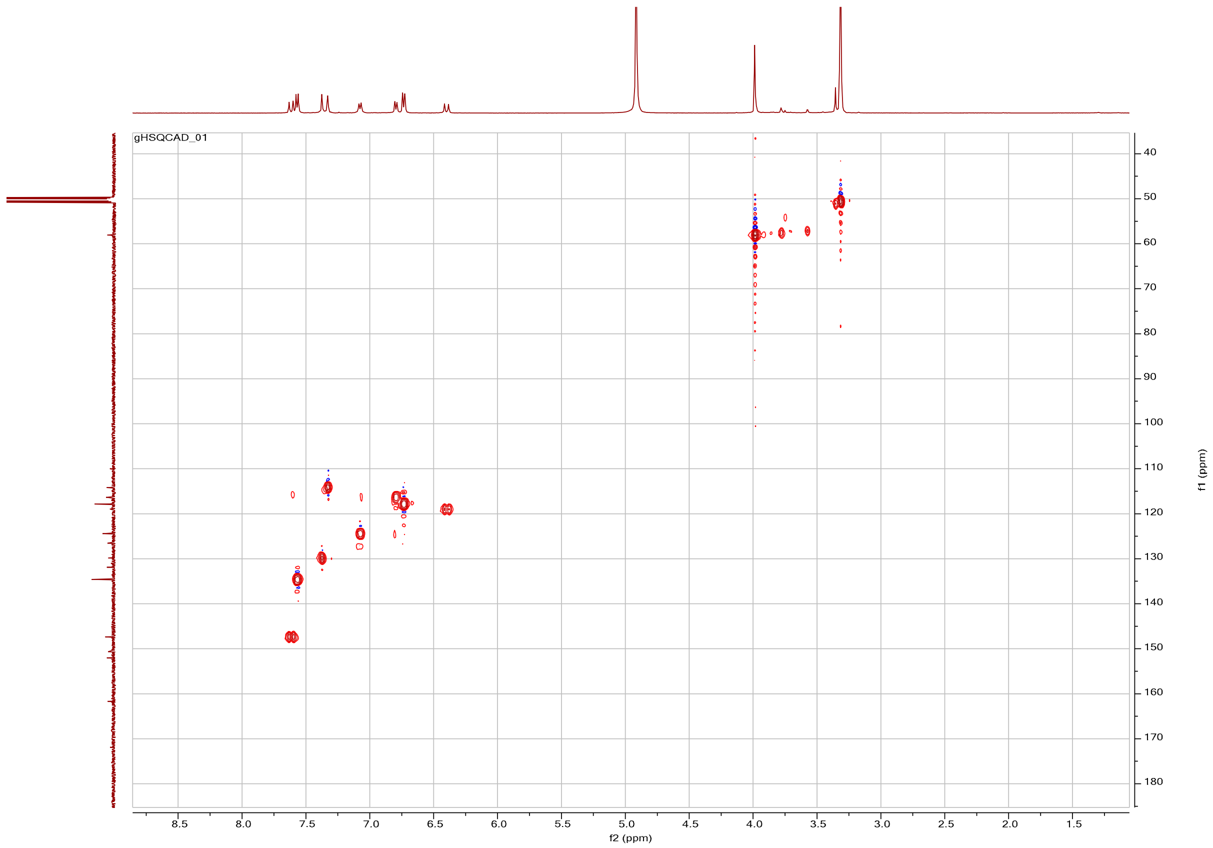

Figure S6. HSQC spectrum of new compound 1 in CH_3OH-*d*$_4$

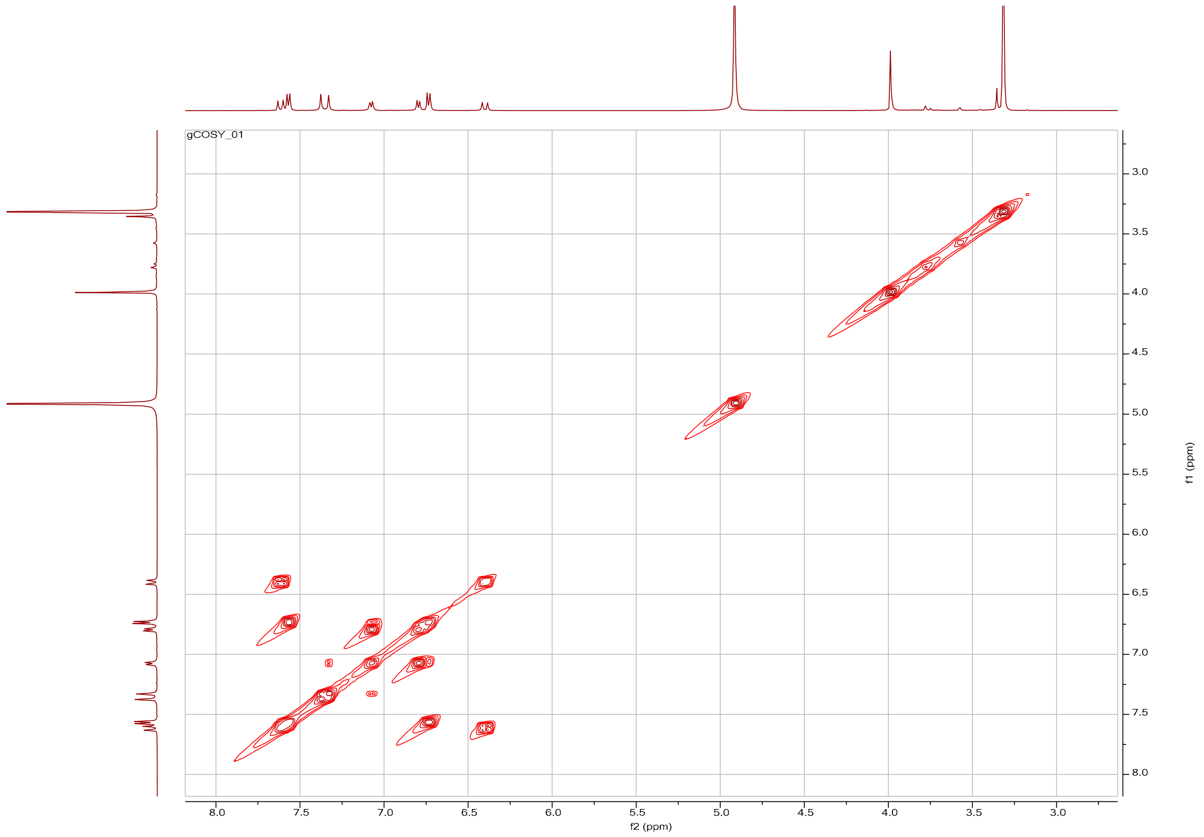

Figure S7. COSY spectrum of new compound 1 in CH_3OH-*d*$_4$

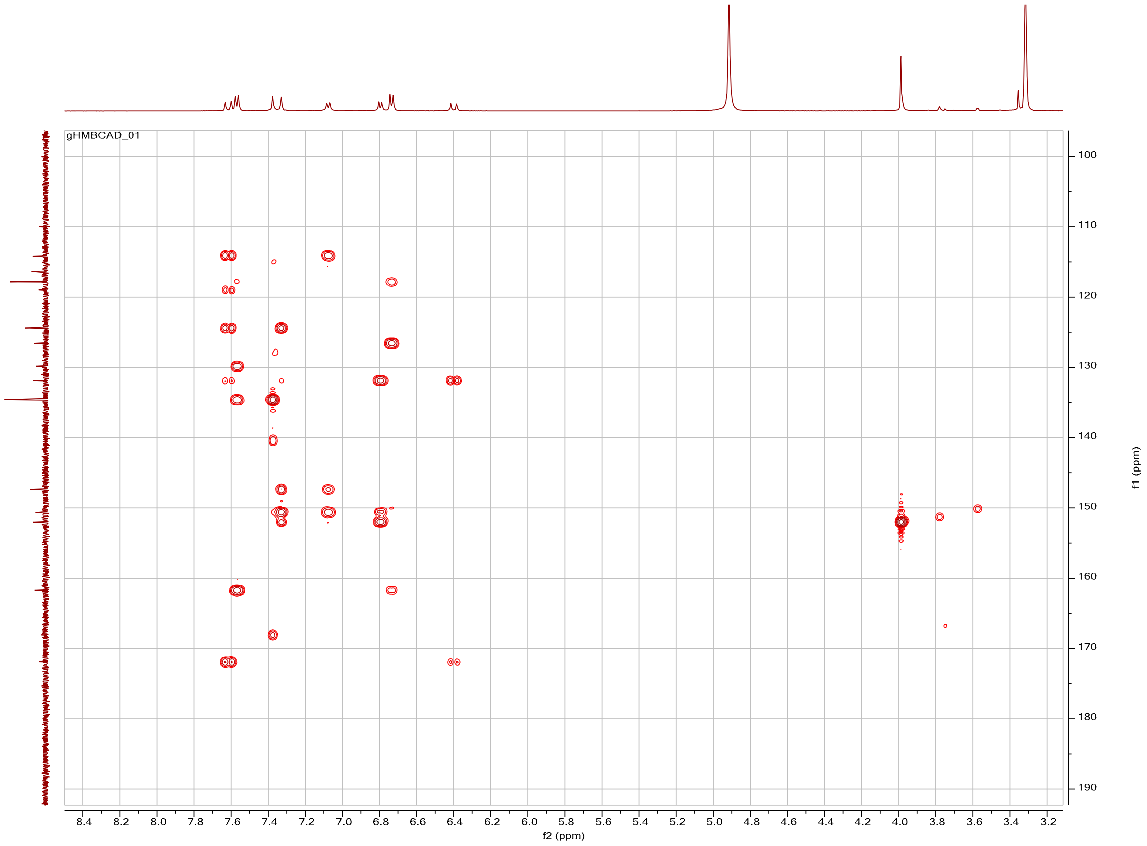

Figure S8. HMBC spectrum of new compound 1 in CH_3OH-*d*$_4$
